# Psychometric properties of the Fluoride Hesitancy Identification Tool (FHIT)

**DOI:** 10.1371/journal.pone.0297188

**Published:** 2024-01-17

**Authors:** Adam C. Carle, Isabella Pallotto, Todd C. Edwards, Richard Carpiano, Darragh C. Kerr, Donald L. Chi

**Affiliations:** 1 James M. Anderson Center for Health Systems Excellence, Cincinnati Children’s Hospital Medical Center, Cincinnati, Ohio, United States of America; 2 Department of Pediatrics, University of Cincinnati College of Medicine, Cincinnati, Ohio, United States of America; 3 Department of Psychology, University of Cincinnati College of Arts and Sciences, Cincinnati, Ohio, United States of America; 4 Department of Health Systems and Population Health, University of Washington School of Public Health, Seattle, Washington, United States of America; 5 School of Public Policy, University of California, Riverside, Riverside, California, United States of America; 6 Department of Oral Health Sciences, University of Washington School of Dentistry, Seattle, Washington, United States of America; Shahid Beheshti University of Medical Sciences School of Dentistry, ISLAMIC REPUBLIC OF IRAN

## Abstract

**Introduction:**

Some caregivers are hesitant about topical fluoride for their children despite evidence that fluoride prevents caries and is safe. Recent work described a five domain model of caregivers’ topical fluoride hesitancy. We developed the Fluoride Hesitancy Identification Tool (FHIT) item pool based on the model. This study sought to evaluate the FHIT’s psychometric properties in an effort to generate a short, simple to score, reliable, and valid tool that measures caregivers’ topical fluoride hesitancy.

**Methods:**

In 2021 and 2022, we conducted an observational, cross-sectional study of caregivers, collecting data from two independent caregiver samples (n_1_ = 523; n_2_ = 612). The FHIT item pool included 33 items. We used confirmatory factor analyses (CFA) to examine whether the FHIT items measured five separate domains as hypothesized and to reduce the number of items. We then fit item response theory (IRT) models and computed Cronbach’s alpha for each domain. Last, we examined the construct validity of the FHIT and evaluated scoring approaches.

**Results:**

After dropping 8 items, CFA supported a five factor model of topical fluoride hesitancy, with no cross-loadings (RMSEA = 0.079; SRMR = 0.057; CFI = 0.98; TLI = 0.98). We further reduced the items to four per domain (20 items total). Marginal alphas showed that the item sets provided reliability of ≥0.90 at hesitancy levels at and above average. The domains correlated more strongly with each other and topical fluoride refusal than with other questions on the survey.

**Discussion:**

Our results support the FHIT’s ability to reliably and validly measure five domains of topical fluoride hesitancy using the average score of the four items in each domain.

## Introduction

Despite evidence that topical fluoride prevents caries and is safe [1–6], caregivers are increasingly hesitant about their children receiving it [7, 8]. Hesitancy may lead to topical fluoride refusal. In turn, insufficient exposure to topical fluoride increases the likelihood of dental caries. Hesitancy may lead to topical fluoride refusal. In turn, insufficient exposure to topical fluoride increases one’s risk for dental caries (tooth decay). Untreated tooth decay may lead to pain, infection, and hospitalization, as well as social and economic consequences, including missed school, poor grades, teasing, and bullying among children and underemployment and lower earnings among adults [7, 9–12]. Despite this, little work has addressed the concept of topical fluoride hesitancy and no published measure of topical fluoride hesitancy currently exists.

Recent work developed a six domain conceptual model of caregivers’ topical fluoride hesitancy [13]. Five domains specifically related to reasons for topical fluoride hesitancy: thinking it is 1) unnecessary, 2) a chemical that should be kept out of their child’s body, and 3) harmful, and feeling 4) there is too much uncertainty about topical fluoride, and 5) distrust about and pressured by dentists and their motives for topical fluoride. We used this model to develop the first tool to measure topical fluoride hesitancy, the Fluoride Hesitancy Identification Tool (FHIT), a pool of 33 items intended to measure the five domains specifically related to hesitancy [14].

The current study sought to evaluate the FHIT item pool’s psychometric properties and create a short, simple to score, reliable, and valid tool to measure caregivers’ topical fluoride hesitancy. First, we used confirmatory factor analyses (CFA) to examine whether the FHIT items measured five separate domains. Second, we used psychometric information to reduce the number of questions and minimize respondent burden while maintaining reliability. Third, we fit item response theory (IRT) models for each domain, delivering a contemporary test theory evaluation of the FHIT’s psychometric properties and a scoring method. Fourth, we computed Cronbach’s alphas to evaluate reliability using classical test theory (CTT). Fifth, we examined the FHIT’s construct validity. And, sixth, we compared different scoring methods.

## Methods

### Design and procedures

We conducted a multisite, observational study. Two independent samples of English-speaking caregivers with at least one child <18 years (*n*_Sample 1_ = 523 and *n*_Sample 2_ = 612) were recruited from November 9^th^ 2020 t0 September 30^th^ 2021 from four urban, pediatric dentistry clinics and social media sites. The majority were recruited from the University of Washington’s Center for Pediatric Dentistry (65% and 93%, samples 1 and 2 respectively).

We administered the FHIT’s 33-item pool within an 85-item REDCap survey [15]. For participants with multiple children, the youngest child was the referent child. Participants provided online consent. Participants provided online consent. Before seeing the survey questions, participants were presented with an informed consent page that contained informed consent information. This page ended with "Click ’Next Page’ to confirm informed consent and begin the survey." All questions were optional and participants could discontinue at any time. All participants who completed a survey were entered into a raffle to win an Apple iPad, 2 Philips Sonicare toothbrushes, or gift cards. The study was approved by the UW Institutional Review Board (STUDY00005790) and conforms to Strobe Guidelines.

### Measures

#### Fluoride Hesitancy Identification Tool

As described in detail elsewhere [14], our team used elicitation interviews, team writing and editing, and cognitive interviews to develop and refine a 33 item pool to measure five domains of topical fluoride hesitancy (Table 1). This item pool demonstrated content validity [14]. For item stems beginning with “I am concerned,” participants indicated the extent to which they were: extremely, somewhat, slightly, or not at all concerned. For all other items, participants indicated the extent to which they strongly agreed, agreed, disagreed, or strongly disagreed with the item stem. All responses were scored 0–3. Zero corresponding to the option indicating the least hesitancy.

**Table 1:** Confirmatory factor analysis (Standardized loadings and factor correlations) and item response theory results [Discrimination (*a*) and location parameters (*b*_*1*_-*b*_*3*_)] for each Fluoride Hesitancy Identification Tool (FHIT) domain.

Domain		Item	Standardized Loadings	*a*	*b*_*1*_	*b*_*2*_	*b*_*3*_
Concern Topical Fluoride is Unnecessary	1	If my child’s teeth are brushed regularly, they do not need TF.	0.93	5.35	-1.04	0.61	1.67
	2	If my child has a healthy diet, they do not need TF.	0.95	6.48	-1.00	0.80	1.73
	3	If my child goes to the dentist regularly, they do not need TF.	0.92	4.86	-1.01	0.87	2.00
	4	If my child gets fluoride from toothpaste, they do not need TF.	0.85	3.26	-1.27	0.67	2.11
	5	I think TF prevents cavities for my child.	0.66	-	-	-	-
	6	I think my child’s teeth can be healthy without TF.	0.66	-	-	-	-
	7	If my child gets fluoride from water, they do not need TF.	-	-	-	-	-
	8	I think my child needs TF.	-	-	-	-	-
Concern Topical Fluoride is Harmful	9	I am concerned TF may cause learning problems for my child.	0.93	4.46	0.73	1.23	1.88
	10	I am concerned TF may cause my child to have autism.	0.88	5.37	1.03	1.42	1.98
	11	I am concerned TF may cause my child to get cancer.	0.91	3.45	0.60	1.21	1.98
	12	I am concerned TF may hurt my child’s IQ.	0.93	5.98	0.92	1.35	1.83
	13	I think TF is harmless for my child.	0.56	-	-	-	-
	14	I am concerned TF may make my child’s teeth look bad.	0.81	-	-	-	-
	15	I think getting TF too often is bad for my child.					
	16	I think TF is unhealthy for my child.	-	-	-	-	-
Feeling Distrust and Pressured by Dentists and their Motives	17	I am concerned that TF is mostly a way for my child’s dentist to make money.	0.79	2.75	1.08	1.65	2.53
	18	I trust what my dentist says about TF.	0.64	1.32	-0.71	2.36	3.85
	19	I am concerned that I am not being told the whole truth about TF.	0.92	2.96	0.40	1.08	1.80
	20	I am concerned that I will feel pressured at my child’s dentist to say yes to TF.	0.84	4.18	0.79	1.35	2.16
	21	I trust that my child’s dentist will give me a choice to say no to TF.	-	-	-	-	-
Feeling Unsure about Topical Fluoride Science	22	I am concerned TF may cause unknown harm in the future.	0.94	2.76	0.30	1.08	1.96
	23	I am concerned about TF because I am not given enough information.	0.81	4.76	0.21	0.96	1.70
	24	I am concerned about TF because some research says it’s not safe.	0.93	5.57	0.32	1.07	1.82
	25	I am concerned about TF because some doctors don’t approve it.	0.90	3.02	0.94	1.43	2.17
	26	I am concerned about TF because I have friends/family who are opposed to it.	0.85	-	-	-	-
	27	I think there is enough proof that TF is safe for my child.	-	-	-	-	-
	28	I think TF has more risks that benefits for my child.	-	-	-	-	-
	29	I am concerned about TF because I do not know how it works.	-	-	-	-	-
Feeling that Topical Fluoride Should be Kept Out of Child’s Body	30	I am concerned about TF because my child already gets too much.	0.86	3.27	0.53	1.16	2.28
	31	I am concerned about TF because my child might swallow it.	0.81	3.19	0.16	1.05	1.95
	32	I am concerned about TF because it is not natural.	0.93	4.46	0.21	0.90	1.83
	33	I am concerned TF may build up in my child’s body.	0.95	3.49	0.41	1.10	1.87
Factor Correlations			Unnecessary	Harmful	Distrust	Unsure	Kept Out
		Concern Topical Fluoride is Unnecessary	1.00				
		Concern Topical Fluoride is Harmful	0.64	1.00			
		Feeling Distrust and Pressured by Dentists and their Motives	0.71	0.96	1.00		
		Feeling Unsure about Topical Fluoride Science	0.65	0.97	0.98	1.00	
		Feeling that Topical Fluoride Should be Kept Out of Child’s Body	0.64	0.95	0.91	0.97	1.00

### Survey questions

We used the following survey questions (analytical values in parentheses) to establish construct validity. A fluoride refusal item asked: “Regarding topical fluoride in general for your child/children, which statement below best describes you?” Caregivers responded: I always say yes (0), I say yes, but I have thought about saying no (1), sometimes I say no (2), most of the time I say no (3), or I always say no (4). A fluoride opposition item asked, “On a scale of 0 to 10 with ‘0’ being ‘not at all opposed’ and ‘10’ being ‘totally opposed’, how opposed are you to topical fluoride for your child/children?” For a dislike item (“I say no or have thought about saying no to topical fluoride because my child/children doesn’t/don’t like getting it”) and a cost item (“I say no or have thought about saying no to topical fluoride because of cost”), caregivers responded: disagree (0) or agree (1). A dental caries likelihood item asked, “How likely is your child to get a cavity?” Caregivers responded: extremely unlikely (0), unlikely (1), likely (2), or extremely unlikely (3). A dental caries severity item asked, “How bad would it be for your child to get a cavity?” Caregivers responded: extremely bad (0), somewhat bad (1), or not that bad (2). Caregivers indicated their child’s: gender (male (0) or female (1)), dental insurance type (private (0) or public (1)), race (White (0) and non-White (1)), and ethnicity (non-Hispanic (0) and Hispanic (1)). And, caregivers indicated their own: gender (male (0) or female (1)), age in years, and annual household income (<$15,000 (0), $15,000 to $25,000 (1), $25,000 to $50,000 (2), $50,000 to $75,000 (3), $75,000 to $100,000 (4), $100,000 to $150,000 (5), and >$150,000 (6)). Although caregivers could select multiple races, we created a white/non-white variable due to small frequencies (Table 2).

**Table 2:** Sample 1 (*n* = 523) and Sample 2 (*n* = 612) demographic characteristics.

	*Sample 1*	*Sample 2*
	Median (IQR) or *n* (%)	Median (IQR) or *n* (%)
**Child demographics**		
Age (years)	6.00 (3–10)	8.00 (5–12)
Gender		
Male	271 (52.12)	297 (49.09)
Female	249 (47.88)	308 (50.91)
Race		
White	294 (57.09)	259 (44.43)
Multiple	110 (21.36)	160 (27.44)
Asian	76 (14.76)	91 (15.61)
Black	35 (6.80)	73 (12.52)
Ethnicity		
Non-Hispanic	434 (83.62)	494 (81.38)
Hispanic	85 (16.38)	113 (18.62)
Insurance type		
Private	267 (51.54)	163 (27.30)
Medicaid or public	214 (41.31)	401 (67.17)
Uninsured	19 (3.67)	6 (1.01)
Other	18 (3.47)	27 (4.52)
**Caregiver demographics**		
Age	41.00 (36.5–46)	42.00 (37–47)
Gender		
Female	407 (82.72)	407 (75.65)
Male	82 (16.67)	128 (23.79)
Other	3 (0.61)	27 (4.52)
Race		
White	303 (63.13)	282 (54.76)
Asian	85 (17.71)	87 (16.89)
Multiple	63 (13.13)	92 (17.86)
Black	29 (6.04)	54 (10.49)
Ethnicity		
Non-Hispanic	434 (89.48)	449 (84.39)
Hispanic	51 (10.52)	83 (15.60)
Education completed		
High school or less	59 (12.02)	98 (18.28)
Some college	119 (24.24)	175 (32.65)
College graduate	130 (26.48)	129 (24.07)
More than a 4-year degree	183 (37.27)	129 (24.07)
Relationship to child		
Mother	413 (79.58)	441 (72.77)
Father	84 (16.18)	147 (24.26)
Other	22 (4.24)	18 (2.97)
Annual household income		
Less than $25,000	65 (13.68)	95 (18.30)
$25,000–$50,000	94 (19.79)	121 (23.31)
$50,000–$75,000	75 (15.79)	100 (19.27)
Greater than $75,000	241 (50.74)	203 (39.11)

### Analytical plan

#### Measurement model

**Fig 1:**
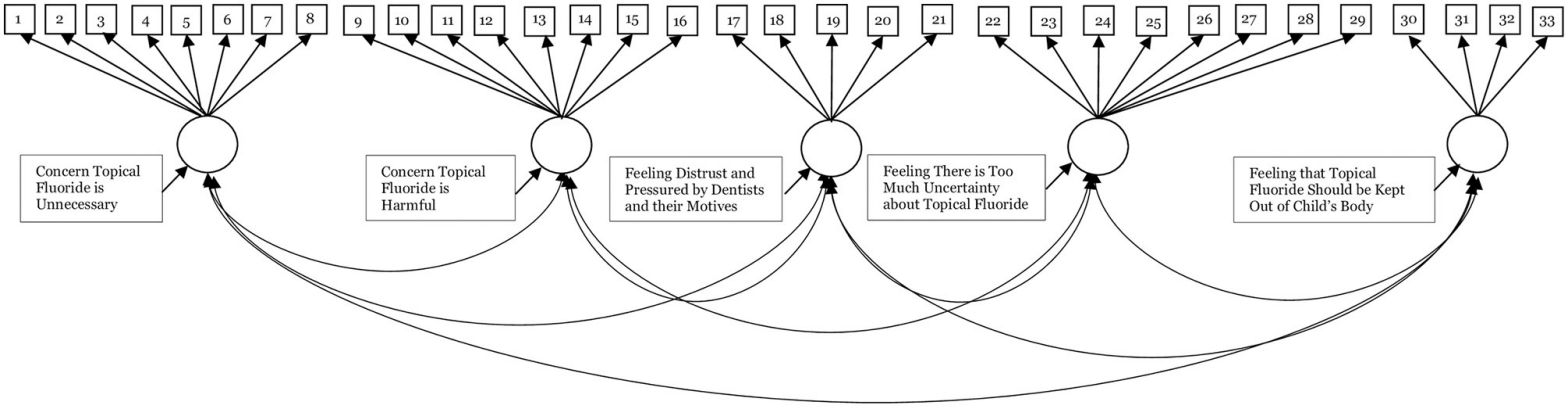
Hypothesized Fluoride Hesitancy Identification Tool (FHIT) measurement model. Note: Circles represent each of the five domains measured by the FHIT. The large square label the domains and are not part of the measurement model. Small squares represent the items. Table 1 gives the item content corresponding to each number. The arrows from the circles to the small squares depict loadings and indicate which items measure a domain. The arrows among the circles depict the correlations among the domains. For simplicity, we do not show all possible measurement parameters.

We used CFA [16, 17] to evaluate whether the items measured the five domains as hypothesized (Fig 1). This also allowed us to examine whether the item sets met some IRT model assumptions [18, 19]. In Sample 1, we used CFA to determine whether the model fit well and, if necessary, modify it. In Sample 2, we evaluated the fit of the model that resulted from the Sample 1 CFA using independent data. We also tested whether a single factor, two factor, or bifactor model fit the data well. We did this to rule out alternative explanations for multidimensionality. We treated root mean square error of approximation (RMSEA) and standardized root mean square residual (SRMR) values <0.05 as demonstrating ideal fit and <0.08 as demonstrating acceptable fit. We considered close fit index (CFI) and Tucker-Lewis index (TLI) values ≥0.95 as indicating ideal fit and values >0.90 and <0.95 as demonstrating acceptable fit. We evaluated fit based on the majority of indices [20, 21]. We used modification indices (MIs), which provide the expected change in fit if a constraint is relaxed [17], to evaluate sources of misfit, like local dependence (LD) and cross-loadings. LD means that the items share a correlation even after accounting for the correlation caused by the domain they measure [16, 17]. Because it can bias parameters and mask the extent to which a response reflects an underlying domain, we dropped one item from each item pair if we found LD. If an item measures more than one domain (cross-loads), it is unclear how much a participant’s response is driven by one domain or another. Thus, we dropped cross-loading items [16, 17].

#### Item reduction

We sought to reduce the items for each domain to minimize respondent burden [22, 23], We used the CFA results and selected the four items that resulted in the greatest information (a measure of reliability) across each domain’s levels [18, 24], We dropped items with the lowest loadings. When two items had similar loadings, we kept the item with thresholds that differed the most from other items. This insured that we selected items that provided reliability across as much of each domain as possible.

#### Item response theory

After establishing the measurement model and reducing items, we fit five separate graded response model (GRM) IRT models for each domain [25] using Sample 2 data and output scores for each domain. We then created marginal reliability curves [26], which translate IRT test information into a Cronbach alpha metric [18, 24]. Unlike CTT, IRT is more realistic and does not assume that reliability is constant [18, 24]. We used a Wald *χ*^2^ and plots comparing observed and expected scores to evaluate fit [27].

#### Classical test theory

We calculated Cronbach’s alphas using each domain’s final four item set and Sample 2 data [28]. We considered values ≥0.90 excellent, <0.90 and ≥0.80 good, and values <0.80 and ≥0.70 adequate.

#### Construct validity

Using Sample 2, we evaluated construct validity by examining the correlations between and across mean levels of each domain and responses to several survey items [16]. We did this three ways. We used 1) structural equation modeling (SEM) [17] to examine the polyserial correlations between and across mean levels of the latent domains and survey items. We also examined polyserial correlations between and across mean levels of 2) domain scores output from IRT and 3) domains scores created by averaging the item responses within a domain and survey items. Last, we examined the extent to which those expressing no hesitancy on each domain reported refusing topical fluoride.

#### Scoring evaluation

Finally, we evaluated whether using a simple scoring approach based on the average of item responses within a domain resulted in substantive conclusions similar to SEM and IRT. SEM is expected to give the best estimate of the relationship between each domain and other variables. However, SEM is analytically complicated, does not provide a score, and can only be used with a relatively large number of individuals. Outputting individuals’ scores using IRT should provide the most accurate method of generating individuals’ scores. However, while IRT provides a score, IRT is still relatively complicated and difficult to implement in clinical settings, especially in real time. An item average-based approach is the least accurate and treats the ordinal responses as if they are interval level measures, yet it is simple to compute in real time in clinical settings and may be more intuitively interpretable because the resulting score is in the metric of the items. While theoretically more accurate than an item average, using IRT scores or item averages when evaluating relationships rather than SEM may lead to biased results [29]. Thus, to evaluate the validity of using the theoretically less accurate but more feasible item average-based approach or alternatively IRT, we compared probability values and standardized parameters and effect sizes across SEM, IRT-based scores, and item average-based scores. We used an average vs. a sum score because this allows calculation even if a caregiver skips an item [17].

We conducted all analyses in Stata [30] and Mplus [31]. All analyses appropriately modeled ordinal data. CFA and SEM used weighted least square mean and variance adjusted estimation and IRT used full information maximum likelihood. Both types allow missing item level data [31].

## Results

### Sample characteristics

Table 2 provides the two independent samples’ demographic characteristics.

### Measurement model

We first fit a five factor model based on the qualitative research. This model did not fit well (RMSEA = 0.10; SRMR = 0.09; CFI = 0.94; TLI = 0.94). MIs indicated LD for eight different item pairs and we dropped one item from each. MIs also revealed that 3 items measured multiple domains and we dropped these items [16, 17]. Finally, “I trust that my child’s dentist will give me a choice to say no to topical fluoride” had a low loading (<0.2), indicating it did not measure the underlying domain well. So, we dropped this item [16, 17].

After dropping these 8 items, the resulting 25-item model, with all remaining items loading on their expected domains, demonstrated sufficient fit (RMSEA = 0.08; SRMR = 0.059; CFI = 0.98; TLI = 0.98). We then tested the fit of the 25 item model in the independent sample. This model fit acceptably: RMSEA = 0.079; SRMR = 0.057; CFI = 0.98; TLI = 0.98. Thus, we considered this the final model. Table 1 presents its standardized loadings and factor correlations. Given that the factor correlations were large (though not problematically large for SEM) [17], we also examined single, two, and a bifactor models. None demonstrated acceptable fit. Therefore, we considered the 25-item, five factor model, the final model.

### Item reduction

As Table 1 shows, for the Concerns Topical Fluoride is Unnecessary and Concerns Topical Fluoride is Harmful domains, we dropped the items with the lowest loadings. For the Feeling Uncertainty about Topical Fluoride domain, we kept the item whose loading equaled 0.81 (vs. 0.85) because the thresholds indicated that this item measured lower hesitancy levels than the other items in the set and thus increased the range of reliability relative to the others. The remaining 2 domains were already at four items each.

### Item response theory

**Fig 2:**
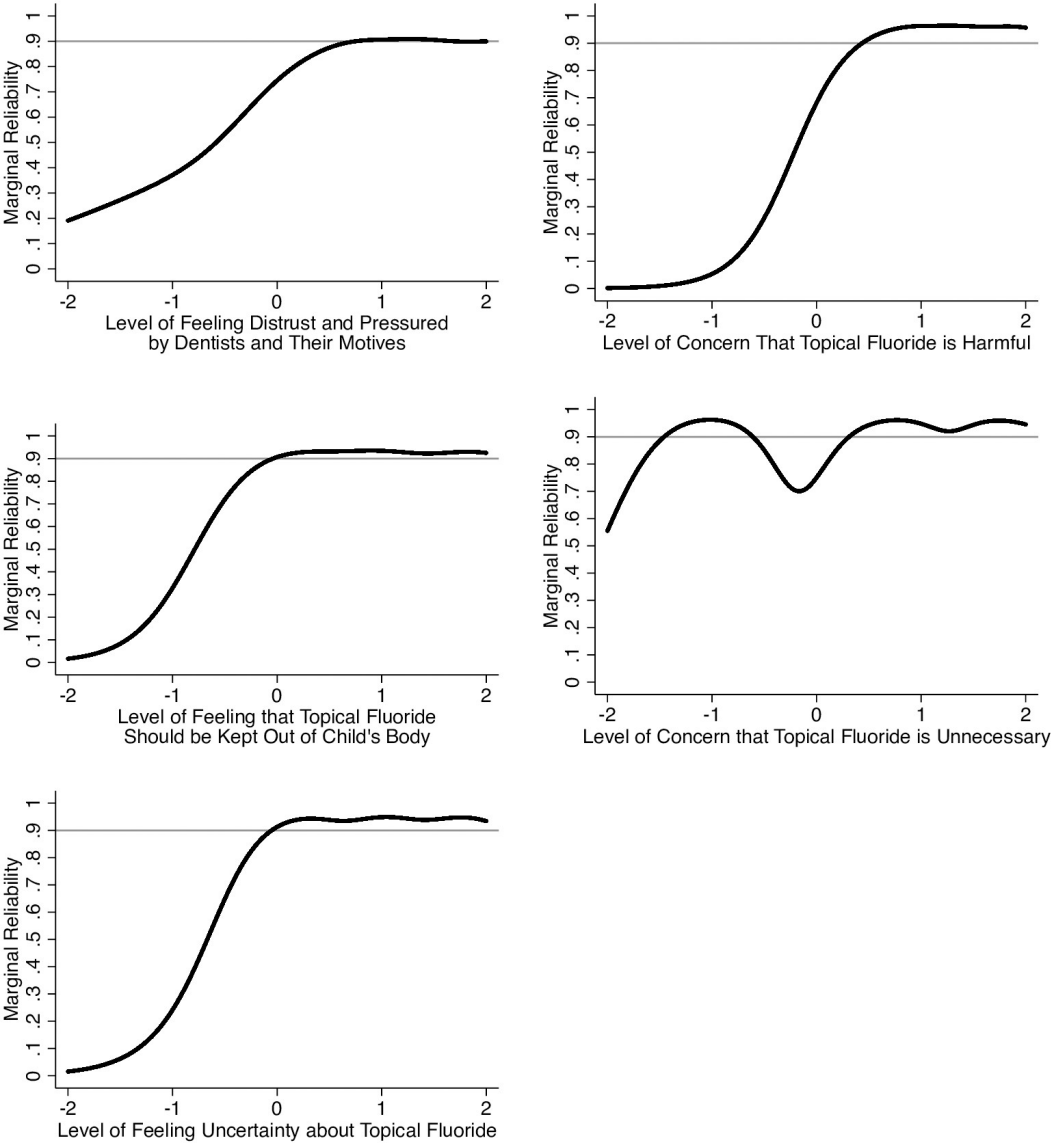
Marginal alpha curves for each domain of topical fluoride hesitancy measured by the Fluoride Hesitancy Identification Tool (FHIT).

Next, we fit GRM for each domain based on each domain’s final four item set. Each fit well. Table 1 lists the parameters. Fig 2 shows the marginal reliability curves. For all domains, reliability is greater than 0.80 at average levels of the latent variable (statistically identified as 0 in IRT) and rapidly and consistently 0.90 and greater for average to high levels of hesitancy. For all domains except Concerns Topical Fluoride is Unnecessary, reliability relatively rapidly decreases at less than average levels.

### Classical test theory

Cronbach’s alpha values equaled 0.92, 0.90, 0.77, 0.88, and 0.89 for Concerns Topical Fluoride is Unnecessary, Concerns Topical Fluoride is Harmful, Feeling Distrust and Pressured by Dentists and their Motives, Feeling Uncertainty about Topical Fluoride, and Feeling that Topical Fluoride Should be Kept Out of Child’s Body, respectively.

### Construct validity

Table 3 presents the validity analyses across all three scoring approaches. Given agreement, we present only the item average findings here. Each of the domains correlated significantly with topical fluoride refusal: *ρ*_Unnecessary_ = 0.64, (*p* < 0.01), *ρ*_Harmful_ = 0.48, (*p* < 0.01), *ρ*_Distrust_ = 0.55, (*p* < 0.01), *ρ*_Uncertainty_ = 0.54, (*p* < 0.01), and *ρ*_Kept Out_ = 0.55, (*p* < 0.01) and opposition to topical fluoride: *ρ*_Unnecessary_ = 0.55, (*p* < 0.01), *ρ*_Harmful_ = 0.43, (*p* < 0.01), *ρ*_Distrust_ = 0.49, (*p* < 0.01), *ρ*_Uncertainty_ = 0.50, (*p* < 0.01), and *ρ*_Kept Out_ = 0.52, (*p* < 0.01). Among caregivers who reported refusing topical fluoride, hesitancy levels did not differ significantly across caregivers that refused due to reasons of cost or a child’s dislike of topical fluoride. Except for Feeling that Topical Fluoride Should be Kept Out of Child’s Body (*ρ*_Kept Out_ = −0.12, *p* = 0.01), the extent to which a caregiver considered it likely that a child would get a cavity was not significantly related to hesitancy levels. However, Concerns Topical Fluoride is Harmful, Feeling Uncertainty about Topical Fluoride, and Feeling that Topical Fluoride Should be Kept Out of Child’s Body were all significantly related to the extent to which a caregiver would consider it bad if a child developed a cavity: *ρ*_Harmful_ = 0.55, *ρ*_Uncertainty_ = 0.55, and *ρ*_Kept Out_ = 0.55 respectively. Decreasing household income was significantly related to increasing hesitancy on each domain: *ρ*_Unnecessary_ = −0.23, (*p* < 0.01), *ρ*_Harmful_ = −0.18, (*p* < 0.01), *ρ*_Distrust_ = −0.17, (*p* < 0.01), *ρ*_Uncertainty_ = −0.22, (*p* < 0.01), and *ρ*_Kept Out_ = −0.19, (*p* < 0.01). Caregiver age was not. Further, as Table 3 shows, while hesitancy levels did not differ significantly across child gender, female caregivers generally reported greater hesitancy levels: *d*_Unnecessary_ = 0.34, (*p* < 0.01), *d*_Harmful_ = 0.31, (*p* < 0.01), *d*_Distrust_ = 032., (*p* < 0.01), and *d*_Kept Out_ = 0.26, (*p* = 0.01). Caregivers of non-White children reported less hesitancy on each domain [*d*_Unnecessary_ = −0.41, (*p* < 0.01), *d*_Harmful_ = −0.39, (*p* < 0.01), *d*_Distrust_ = −034., (*p* < 0.01), *d*_Uncertainty_ = −0.39, (*p* < 0.01), and *d*_Kept Out_ = −0.39, (*p* < 0.01)] than caregivers of White children. However, caregivers of Hispanic children did not report significantly different levels of hesitancy in any domain relative to caregivers of non-Hispanic children. Finally, caregivers of children with public dental insurance reported significantly lower levels of hesitancy due to Concerns Topical Fluoride is Harmful (*d*_Harmful_ = -0.20, *p* = 0.03) and Feeling Uncertainty about Topical Fluoride (*d*_Uncertainty_ = −0.28, *p* = < 0.01) than caregivers of children with private insurance but no other domains.

**Table 3:** Effect size mean differences, standardized correlations, and probabilities across Fluoride Hesitancy Identification Tool (FHIT) domains, survey variables, and scoring approaches.

	Mean Difference Relationships (Effect Size Metric)						Correlational Relationships (Correlation Metric)					
	d	*p*	d	*p*	d	*p*	ρ	*p*	ρ	*p*	ρ	*p*
	Item Mean-Based Score		IRT-Based Score		SEM-Based		Item Mean-Based Score		IRT-Based Score		SEM-Based	
	Child Gender						Refusal					
Unnecessary	0.09	0.29	0.08	0.32	0.09	0.33	0.64	0.00	0.67	0.00	0.69	0.00
Harmful	0.12	0.16	0.08	0.31	0.12	0.25	0.48	0.00	0.53	0.00	0.65	0.00
Distrust and Pressure	0.08	0.35	0.07	0.37	0.08	0.45	0.55	0.00	0.58	0.00	0.73	0.00
Uncertainty	0.14	0.10	0.15	0.08	0.17	0.09	0.54	0.00	0.59	0.00	0.67	0.00
Kept Out of Body	0.11	0.20	0.14	0.09	0.14	0.16	0.55	0.00	0.59	0.00	0.68	0.00
	Child Dental Insurance Type						Opposition					
Unnecessary	-0.16	0.08	-0.17	0.06	-0.18	0.07	0.54	0.00	0.54	0.00	0.57	0.00
Harmful	-0.20	0.03	-0.24	0.01	-0.30	0.02	0.43	0.00	0.47	0.00	0.59	0.00
Distrust and Pressure	-0.16	0.09	-0.15	0.10	-0.19	0.12	0.49	0.00	0.50	0.00	0.66	0.00
Uncertainty	-0.28	0.00	-0.29	0.00	-0.33	0.00	0.50	0.00	0.53	0.00	0.60	0.00
Kept Out of Body	-0.18	0.06	-0.20	0.03	-0.22	0.05	0.52	0.00	0.55	0.00	0.64	0.00
	Child Ethnicity						Cavity Likelihood					
Unnecessary	-0.03	0.80	-0.03	0.75	0.03	0.77	-0.06	0.22	-0.05	0.29	-0.06	0.20
Harmful	-0.09	0.43	-0.04	0.73	0.09	0.52	0.08	0.10	0.10	0.03	0.11	0.06
Distrust and Pressure	0.01	0.93	0.05	0.63	-0.02	0.86	0.03	0.54	0.05	0.25	0.05	0.32
Uncertainty	-0.09	0.43	-0.01	0.92	0.06	0.63	0.08	0.09	0.09	0.06	0.10	0.04
Kept Out of Body	0.09	0.40	0.11	0.31	-0.10	0.41	0.12	0.01	0.13	0.00	0.14	0.00
	Child Race						How bad would it be for your child to get a cavity?					
Unnecessary	-0.41	0.00	-0.41	0.00	-0.45	0.00	0.05	0.29	0.04	0.36	0.05	0.27
Harmful	-0.39	0.00	-0.49	0.00	-0.58	0.00	-0.13	0.01	-0.15	0.00	-0.17	0.00
Distrust and Pressure	-0.34	0.00	-0.37	0.00	-0.45	0.00	-0.07	0.14	-0.08	0.08	-0.09	0.11
Uncertainty	-0.39	0.00	-0.40	0.00	-0.48	0.00	-0.12	0.01	-0.12	0.01	-0.14	0.01
Kept Out of Body	-0.39	0.00	-0.39	0.00	-0.47	0.00	-0.12	0.01	-0.12	0.01	-0.15	0.01
	Caregiver Gender						Household Income					
Unnecessary	0.34	0.00	0.33	0.00	0.36	0.00	-0.23	0.00	-0.23	0.00	-0.25	0.00
Harmful	0.31	0.00	0.31	0.00	0.37	0.00	-0.18	0.00	-0.21	0.00	-0.26	0.00
Distrust and Pressure	0.32	0.00	0.28	0.01	0.37	0.00	-0.17	0.00	-0.14	0.00	-0.19	0.00
Uncertainty	0.15	0.14	0.10	0.32	0.15	0.21	-0.22	0.00	-0.20	0.00	-0.26	0.00
Kept Out of Body	0.26	0.01	0.23	0.02	0.28	0.02	-0.19	0.00	-0.20	0.00	-0.23	0.00
	Dislike						Caregiver Age					
Unnecessary	0.21	0.19	0.19	0.24	-	-	-0.04	0.31	-0.05	0.22	-0.05	0.32
Harmful	0.22	0.20	0.11	0.53	-	-	0.01	0.77	0.01	0.91	0.02	0.68
Distrust and Pressure	-0.07	0.69	-0.17	0.30	-	-	-0.01	0.83	-0.03	0.45	-0.02	0.72
Uncertainty	-0.13	0.45	-0.25	0.15	-	-	-0.05	0.26	-0.05	0.22	-0.06	0.24
Kept Out of Body	-0.18	0.28	-0.25	0.14	-	-	-0.02	0.64	-0.03	0.56	-0.03	0.60
	Cost											
Unnecessary	0.25	0.19	0.24	0.20	-	-	-	-	-	-	-	-
Harmful	0.38	0.05	0.34	0.08	-	-	-	-	-	-	-	-
Distrust and Pressure	0.15	0.43	0.24	0.21	-	-	-	-	-	-	-	-
Uncertainty	0.06	0.75	-0.05	0.79	-	-	-	-	-	-	-	-
Kept Out of Body	-0.10	0.62	-0.16	0.41	-	-	-	-	-	-	-	-

We also examined the extent to which those expressing no hesitancy on a domain, reported refusing topical fluoride. Among those with an item average-based score of zero (no hesitancy) on the Concerns Topical Fluoride is Unnecessary, Concerns Topical Fluoride is Harmful, Feeling Distrust and Pressured by Dentists, Feeling Uncertainty about Topical Fluoride, and Feeling that Topical Fluoride Should be Kept Out of Child’s Body, 100%, 95%, 98%, 98%, and 97%, respectively, reported never refusing topical fluoride.

### Scoring evaluation

As Table 3 shows, effect sizes (*d*) and standardized parameter estimates (*ρ*) across approaches were similar. The item average- and IRT score-based approaches occasionally underestimated the size of relationships to a small degree relative to SEM. With only two exceptions, the three approaches agreed perfectly with respect to the probability that a given effect occurred by chance. The IRT-based approach probability for the relation between the extent to which a caregiver considered it likely that a child would get a cavity and Concerns that Topical Fluoride is Harmful was less than 0.05 (*p* = 0.03) while the SEM probability value was greater than 0.05 (*p* = 0.06). Conversely, the item average- and IRT score-based approach probabilities for the relation between the extent to which a caregiver considered it likely that a child would get a cavity and Feeling Uncertainty about Topical Fluoride Science were greater than 0.05 (*p* = 0.08 and 0.10 respectively), while the SEM value was less than 0.05 (*p* = 0.04).

## Discussion

This study sought to evaluate the FHIT item pool’s psychometric properties and establish a short, simple to score, reliable, and valid method of measuring domains of caregivers’ topical fluoride hesitancy for their children. Our results support the reliability and validity of a 20-item FHIT to measure five domains of topical fluoride hesitancy using the average of the four items measuring each domain.

CFA supported the FHIT’s internal validity. Caregivers’ fluoride hesitancy appears to be comprised of five domains: feeling that topical fluoride is 1) unnecessary, 2) a chemical that should be kept out of their child’s body, and 3) harmful, and feeling 4) there is too much uncertainty about topical fluoride, and 5) distrust about and pressured by dentists and their motives for topical fluoride. The FHIT items measure one and only one domain each. While the domains are correlated, a caregiver could conceptually be high on one and low on others. Efforts to understand, prevent, and address topical fluoride hesitancy should consider an individual’s reported values for each domain and their joint impact.

CTT and IRT supported the reliability of each FHIT domain. Cronbach’s alphas suggested the domains generally provided excellent reliability. IRT showed that the items provided high reliability at and above average hesitancy levels but that reliability tended to decrease relatively rapidly at lower than average levels of hesitancy. While a uniformly high level would be ideal, the decrease in reliability is unlikely to be problematic in contexts where the FHIT is likely to be used. Clinicians using the FHIT to identify individuals with topical fluoride hesitancy do not need to discriminate well among those with low hesitancy. Similarly, researchers evaluating efforts to address hesitancy will be most interested in the effect of these efforts among individuals with elevated hesitancy. The FHIT is well suited for these tasks.

Our results also support construct validity. Except for topical fluoride refusal, the domains correlated more strongly with each other than with other survey questions. And, as expected, the correlations between domain scores and refusal of and opposition to topical fluoride were large generally and relative to the size of other variables’ correlations with the domain scores. In addition, as expected, domain scores did not differ significantly across a child’s gender but did differ across caregiver’s gender. While we did not have specific hypotheses for other variables, validity was supported by the fact that the domains significantly correlated with (or levels significantly differed across) some variables (e.g., household income) but not others (e.g., caregiver’s age).

With respect to scoring, our findings support the validity of an item average-based score. This score is created for each domain’s four item set by 1) assigning values of 0–3 to participants’ responses for each item, with 0 corresponding to the option indicating the least hesitancy, and 2) taking the average of the responses. The validity of this approach is important because users may not have access or ability to use SEM or IRT. Further, because this approach is in the metric of the item responses, an item average-based score allows interpretation relative to the item responses.

Finally, our study provides the first quantitative evidence that topical fluoride hesitancy is multifactorial. Previous qualitative work has suggested the complex nature of topical fluoride hesitancy [13, 32] but it was not clear to what extent that complexity represented a set of underlying domains. Our findings indicate that five correlated domains reflect parents’ hesitancy about topical fluoride. Thus, while dentists may assume that caregivers are hesitant due to lack of knowledge [33], our results suggest that education alone will likely not reduce hesitancy across these domains. Future efforts should evaluate the predictors of the different domains of topical fluoride hesitancy and develop and evaluate interventions to reduce topical fluoride hesitancy and refusal.

### Limitations

Before concluding, we note some weaknesses. Participants were from mainly from a university-based dental school in Seattle and the FHIT is only available in English. Yet, the study’s strengths, which include independent samples, a confirmatory rather than exploratory approach, and evaluating a range of approaches to examine construct validity and support scoring, outweigh these weaknesses.

## Conclusion

In conclusion, our study supports the reliability and validity of the 20-item FHIT as a measure of five domains of caregiver’s reasons for hesitance to topical fluoride.
